# Significant Increase in the Prevalence of Multiple Sclerosis in Iran in 2011

**Published:** 2014-03

**Authors:** Sadegh Izadi, Alireza Nikseresht, Maryam Sharifian, Mohammad Ali Sahraian, Alireza Hamidian Jahromi, Mohammad Aghighi, Alireza Heidary

**Affiliations:** 1 Department of Neurology, Motahari Clinic, Shiraz University of Medical Sciences, Shiraz, Iran;; 2 Student Research Committee, Shiraz University of Medical Sciences, Shiraz, Iran;; 3 Department of Neurology, Tehran University of Medical Sciences, Tehran, Iran;; 4 Department of Surgery, Louisiana State University Health Sciences Center, Shreveport, LA, USA;; 5 Department of Transplantation and Special Diseases, Ministry of Health and Medical Education, Tehran, Iran

## Dear Editor,

Multiple sclerosis (MS) is a chronic demyelinating disease of the central nervous system and usually affects young women. It is the second most common cause of neurological disability in young adults after trauma.^1^^,^^2^ Previously, Iran was considered a low prevalence area, but recent investigations have shown that the prevalence of MS in Iran has increased significantly.^3^ This increasing pattern in the rate of MS may have several causes; however, changes in lifestyle and new and advanced diagnostic methods are regarded as the most important causes.

Unfortunately, Iran does not have a national registry for patients with MS. Nevertheless, there is a national computerized registration system which holds the information of every patient with MS in the country that has registered and receives beta interferon medication from the Ministry of Health and Medical Education (MOHME). The Iranian government covers a considerable percentage of the treatment costs for patients with MS receiving beta interferon according to this registry. 

Although studying the data on this group of patients does not yield precise and comprehensive information on all patients with MS in Iran, even an evaluation of these data demonstrates that the prevalence rate has increased significantly. 

Our study was conducted based on the data derived from the latest report of Iran’s MOHME about the patients who registered to receive beta interferon in Iran. We collected data from the MS Registry Database of the different provinces of Iran which had been sent to the MOHME by the end of year 2011. Data analysis was performed using the Statistical Package for Social Sciences (SPSS) version 17 under the supervision of an expert epidemiologist. In December 2011, the Iranian MOHME Registry listed 34,605 MS patients in Iran. Seventy-seven percent of whom were women. Given that Iran’s population in 2011 was 75,600,000, the prevalence rate of MS was calculated as 45/100,000 of population.^4^ Seventy percent of these patients were between 20-40 years of age. The maximum prevalence rate (80 per 100,000 population) was seen in Isfahan province, located in the central part of Iran. The minimum prevalence rate (6 per 100,000 population) was seen in Sistan-Baluchestan province, located in the southeast part of Iran. This province has a warm and dry climate.

Ten years ago, Kalanie et al.^5^ reported 200 patients with a definite diagnosis of MS in Iran. In 2005, Etemadifar  et al.^3^ reported a prevalence rate of 35.5 per 100,000 of population, with a female/male ratio of 3.6. Two years later, again in Isfahan province, another study reported a prevalence rate of 43.8 per 100,000 of population.^7^ In 2010, a study was performed by Sahraian et al.^6^ in the capital city of Iran (Tehran). The authors estimated that the point prevalence of MS in Tehran was at least 51.9 per 100,000 of population with a female/male ratio of about 3.12.

In the present study, although we only considered patients who received beta interferon, it seems that the prevalence rate of MS has increased both at national and provincial levels. These rates are similar to what is seen in western countries.^6^ Interestingly, this rise in the prevalence rate of MS has also been seen in some neighboring countries of Iran such as Kuwait.^8^ There are some explanations for this increasing pattern in the prevalence of MS in the Middle East and also Iran. One of the most important causes may be the age of the population in Iran. Iran has a very young population, with the majority of the population in Iran being in the age range of 15-30 years. The fact that the disease is more prevalent in young adults means that age may be an important factor for the rise in the prevalence rate. Furthermore, it has been previously demonstrated vitamin D deficiency is very prevalent in Iran. This may be the consequence of the increase in the number of people living in apartments, the rise in the consumption of cosmetics and sunscreen creams, or exacerbation of air pollution especially in industrial areas such as Isfahan, Tehran, and Fars provinces, all of which have the highest rates of MS in Iran. The relation between vitamin D deficiency and MS has been reported by several studies. Accordingly, the increase in the prevalence of vitamin D deficiency may be another explanation for the rise in the prevalence rate of MS in Iran.^3^

In addition to these facts, it is important to consider the effect of new diagnostic methods. Improvement in diagnostic methods has led to a rise in the detection of more patients with MS, which is another contributing factor to the increase in the prevalence rate of MS worldwide and also in Iran. 

To sum up, the current study suggests that Iran has a moderate to high MS prevalence rate, with a recent sharp increase in this rate. 
